# Exploratory Investigation of the Plasma Proteome Associated with the Endotheliopathy of Trauma

**DOI:** 10.3390/ijms23116213

**Published:** 2022-06-01

**Authors:** Joseph D. Krocker, Kyung Hyun Lee, Hanne H. Henriksen, Yao-Wei Willa Wang, Erwin M. Schoof, Sigurdur T. Karvelsson, Óttar Rolfsson, Pär I. Johansson, Claudia Pedroza, Charles E. Wade

**Affiliations:** 1 Center for Translational Injury Research, Department of Surgery, McGovern Medical School at the University of Texas Health Science Center at Houston, Houston, TX 77030, USA; yao-wei.w.wang@uth.tmc.edu (Y.-W.W.W.); charles.e.wade@uth.tmc.edu (C.E.W.); 2 Center for Clinical Research and Evidence-Based Medicine, Department of Pediatrics, McGovern Medical School at The University of Texas Health Science Center at Houston, Houston, TX 77030, USA; kyung.hyun.lee@uth.tmc.edu (K.H.L.); claudia.pedroza@uth.tmc.edu (C.P.); 3 Center for Endotheliomics CAG, Department of Clinical Immunology, Copenhagen University Hospital, Rigshospitalet, 2200 Copenhagen, Denmark; hanne.hee.henriksen@regionh.dk; 4 Department of Biotechnology and Biomedicine, Technical University of Denmark, 2800 Lyngby, Denmark; erws@dtu.dk; 5 Center for Systems Biology, University of Iceland, 101 Reykjavik, Iceland; stk13@hi.is (S.T.K.); ottarr@hi.is (Ó.R.); 6 Center for Endotheliomics CAG, Department of Clinical Immunology, Rigshospitalet, & Department of Clinical Medicine, University of Copenhagen, 2200 Copenhagen, Denmark; per.johansson@regionh.dk

**Keywords:** proteomics, trauma, syndecan-1, soluble thrombomodulin, endothelium, inflammation, sympathetic, damage-associated molecular patterns, complement, coagulopathy

## Abstract

Background: The endotheliopathy of trauma (EoT) is associated with increased mortality following injury. Herein, we describe the plasma proteome related to EoT in order to provide insight into the role of the endothelium within the systemic response to trauma. Methods: 99 subjects requiring the highest level of trauma activation were included in the study. Enzyme-linked immunosorbent assays of endothelial and catecholamine biomarkers were performed on admission plasma samples, as well as untargeted proteome quantification utilizing high-performance liquid chromatography and tandem mass spectrometry. Results: Plasma endothelial and catecholamine biomarker abundance was elevated in EoT. Patients with EoT (*n* = 62) had an increased incidence of death within 24 h at 21% compared to 3% for non-EoT (*n* = 37). Proteomic analysis revealed that 52 out of 290 proteins were differentially expressed between the EoT and non-EoT groups. These proteins are involved in endothelial activation, coagulation, inflammation, and oxidative stress, and include known damage-associated molecular patterns (DAMPs) and intracellular proteins specific to several organs. Conclusions: We report a proteomic profile of EoT suggestive of a surge of DAMPs and inflammation driving nonspecific activation of the endothelial, coagulation, and complement systems with subsequent end-organ damage and poor clinical outcome. These findings support the utility of EoT as an index of cellular injury and delineate protein candidates for therapeutic intervention.

## 1. Introduction

Unintentional injury remains the third leading cause of death in the United States [1] and persists as the leading cause of mortality for individuals aged one to 44 years old [2]. The economic burden generated by trauma exceeds 189 billion dollars annually [2]. While there have been some advancements in the mitigation of early mortality in traumatic injury, progress has proven to be more difficult in improving long-term survival [3].

The endotheliopathy of trauma (EoT), a form of shock-induced endotheliopathy (SHINE), has been postulated to represent a major component driving outcomes in trauma [4,5,6]. Endotheliopathy signifies dysfunction of the endothelium as well as disruption of its overlying glycocalyx layer. The vascular endothelium is responsible for maintaining the permeability barrier, in part through structural components including adherens junctions [7]. The glycocalyx is composed of proteoglycans and glycoproteins fixed to the endothelial cell surface which serve as a scaffold for soluble proteins and other soluble components [8]. The unique composition of these glycosaminoglycans dictates interactions with extracellular ligands and modulates systemic inflammatory and coagulation profiles [8,9,10]. Some consider this intricate relationship between blood and the endothelium an organ system unto itself [7,11]. Endotheliopathy represents a contributor to ongoing physiologic dysfunction common to all forms of shock and a target for therapeutic intervention [6].

Circulating biomarkers of endotheliopathy have been utilized to reveal an inextricable link between the condition of the endothelium, the glycocalyx, and the physiology of the trauma patient. Trauma-induced endotheliopathy has been implicated in permeability barrier attenuation [12,13,14], sympathoadrenal hyperactivation [15,16,17,18,19,20], coagulopathy [17,21,22,23,24,25], hypoxia and metabolic derangement [17,19,26]. Endothelial dysfunction following injury has been associated with increased injury severity [17,19], multiple organ failure [26], increased intensive care unit stay [17,18], and increased mortality [17,18,19,27]. Despite these associations, the underlying etiology and clinical utility of endotheliopathy in trauma have not yet been fully elucidated. Proteomic profiling provides a more comprehensive representation of the systemic response to injury [28,29]. Herein, we investigate the plasma proteome of EoT in order to gain new insight into the molecular mechanisms underlying endothelial and glycocalyx dysfunction and to identify new targets for therapeutic intervention.

## 2. Methods

### 2.1. Patient Population

Patients presenting to the emergency department requiring the highest level of trauma activation between March 2012 and February 2018 at Hermann Memorial Hospital, a level-1 trauma center in Houston, Texas, were included in an ongoing prospective universal protocol and considered eligible for the study. The highest level of trauma activation requires at least one of the following: systolic blood pressure < 90 mm Hg, heart rate > 120 beats per minute, respiratory rate > 29 or <10 breaths per minute, Glasgow coma score < 10, intubation, prehospital blood product administration, uncontrolled external hemorrhage, penetrating injury to the head, neck, torso or groin, amputation proximal to the wrist or ankle, paraplegia, quadriplegia, pelvic fracture or two or more long bone fractures. Study subjects were selected retrospectively from this universal cohort by stratified randomization based on injury severity score and mechanism of injury to achieve a balanced patient population for this exploratory analysis. Exclusions included prisoners, pregnant patients, those less than 16 years of age, and those who declined to consent to the study. Traumatic brain injury (TBI) influences patient outcomes through mechanisms that are not necessarily related to endotheliopathy within the systemic vasculature. For this reason, subjects with a head abbreviated injury score > 2 representing moderate or severe traumatic brain injury were excluded, including TBI subjects with multi-trauma. Patient demographics, clinical information, and outcome data were obtained from the institutional trauma registry or medical record extraction by research personnel. Consent from patients or legally authorized representatives was obtained within 72 h of admission. A waiver of consent was procured for patients who expired, were discharged within 24 h of admission, or were lacking decision-making capacity without a surrogate decision-maker following at least three attempts to acquire consent over 72 h. This prospective, observational study was approved by the McGovern Medical School at UTHealth institutional review board (HSC-GEN-12-0059). Study subjects were randomly selected retrospectively based on injury severity score and mechanism of injury.

### 2.2. Sample Collection and Processing

Admission blood samples were collected for each patient upon emergency department arrival in 3.2% sodium citrate and ethylenediaminetetraacetic acid (EDTA) tubes and inverted several times. Samples were centrifuged at 1800× *g* at 5 °C for 10 min twice. The plasma layer was isolated, aliquoted, and stored at −80 °C.

### 2.3. Enzyme-Linked Immunosorbent Assay (ELISA)

The soluble biomarkers of sympathoadrenal activation (epinephrine and norepinephrine), endothelial glycocalyx shedding (Syn-1), and endothelial damage (soluble thrombomodulin; sTM) were measured by immunoassays according to the manufacturer’s recommendation (2-CAT ELISA, Labor Diagnostica Nord GmbH & Co. KG, Nordhorn, Germany and Diaclone Nordic Biosite, Copenhagen, Denmark) using citrate plasma.

### 2.4. High Performance Liquid Chromatography and Tandem Mass Spectrometry (MS)

#### 2.4.1. Sample Preparation and Digestion

Sample preparation and digestion were performed without a high abundance protein depletion step using EDTA plasma according to methods previously published by Kulak et al. [30]. Briefly, plasma proteins were denatured using 20 µL of buffer consisting of 6 M guanidinium hydrochloride, 10 mM TCEP (Tris(2-carboxyethyl)phosphine hydrochloride; Sigma), 40 mM CAA (40 mM 2-Chloroacetamide; Sigma, Burlington, MA, USA), and 50 mM HEPES (N-(2-Hydroxyethyl)piperazine-N′-(2-ethanesulfonic acid), 4-(2-Hydroxyethyl)piperazine-1-ethanesulfonic acid; Sigma) at pH 8.5. Samples were then boiled at 95 °C for 5 min, and subsequently sonicated on high for 30 s five times in a Bioruptor sonication water bath (Diagenode, Denville, NJ, USA) at 4 °C. Protein concentration was determined by a Bradford assay (Sigma). Protein digestion was then performed with 5 µg of protein. Samples were diluted 1:3 in a solution consisting of digestion buffer (10% acetonitrile and 50 mM HEPES at pH 8.5) and Endoproteinase Lys-C (MS grade; Wako, Neuss, Germany) was added in a 1:50 enzyme-to-protein ratio. Samples were incubated at 37 °C for 4 h. A subsequent 1:10 dilution was completed with digestion buffer (10% acetonitrile and 50 mM HEPES at pH 8.5) and a 1:100 enzyme-to-protein ratio of trypsin (MS grade; Promega, Madison, WI, USA) was added. Samples were then incubated overnight at 37 °C. Enzyme activity was quenched with the addition of 2% trifluoroacetic acid (TFA) to create a final concentration of 1%. Subsequently, 500 ng of sample was loaded onto EvoSep stagetips according to the manufacturer’s protocol.

#### 2.4.2. Data Acquisition

Samples underwent protein analysis utilizing an EvoSep One instrument on the default “30 samples per day” setting. A Q-Exactive HF-X instrument (ThermoFisher Scientific, Waltham, MA, USA) with a DD-MS2 top28 method was used for analysis following protein elution over a 44 min gradient. A resolution of 60,000 with an automatic gain control (AGC) target of 3 × 10^6^ or a maximum injection time of 50 ms and a scan range of 350–1850 m/z was used for the recording of the full mass spectrometry (MS) spectra. A resolution of 15,000 with an AGC target value of 1 × 10^5^ or a maximum injection time of 22 ms with a normalized collision energy of 28 and an intensity threshold of 2 × 10^4^ was used for MS2 spectra acquisition. Exclusions included ions with a charge state of <2, >6, and unknown with a dynamic exclusion at 30 s. MS consistency was verified using complex cell lysate quality control standards and chromatography was evaluated for reproducibility.

#### 2.4.3. Protein Quantitation

Raw MS data were analyzed using Proteome Discoverer 2.2. Processing and consensus analysis was completed with label-free quantitation. Spectra protein matching was completed using UniProt and the 9606 Human database. Dynamic modifications included oxidation (methionine), deamidation (asparagine, glutamine), and acetyl groups located on N-termini. Cysteine carbamidomethyl was considered a static modification. Protein quantification was completed using the built-in Minora Feature Detector with a false discovery rate (FDR) filter of 1% and a built-in T-test to evaluate statistical significance.

### 2.5. Statistical Analysis

#### 2.5.1. Clinical Variables and ELISA

EoT was defined as a plasma Syn-1 concentration of ≥40 ng/mL [17,18,31,32]. Comparison of EoT vs non-EoT groups for clinical variables and ELISA quantification consisted of a Wilcoxon rank-sum test for continuous variables, a Fisher exact test for categorical variables, and an Armitage trend test for ordinal variables.

#### 2.5.2. MS Protein Analysis

Following MS protein quantification at an FDR threshold of 0.01, proteins with excessive missing values (>30%) were excluded, as were proteins with ≥10 extreme outliers (i.e., values above or below $Q3\pm 3\times \left(Q3-Q1\right)$, where Q1 and Q3 are the first and third quartile, respectively). Patients without adequate plasma samples for proteomic analysis were excluded. Values below a detection limit (initially recorded as 0) were replaced by 10% of the smallest non-zero value of each protein. After applying a $log_{2}$-transformation, the values of each protein were then standardized (centered at 0 with variance 1).

#### 2.5.3. Identification of Differentially Expressed Proteins

Identification of differentially expressed proteins between EoT and non-EoT groups was initiated with a two-sided *t*-test to obtain a test statistic and a raw *p*-value of each protein. To account for high dimensional data, the raw *p*-values were then adjusted using the method of Benjamini and Hochberg at 5% to control the false discovery rate [33]. Fold change was calculated as *log*_2_ of the ratio of a mean protein level in the EoT group to that of the non-EoT group. Analysis of differentially expressed proteins was completed using the QIAGEN Ingenuity Pathway Analysis (IPA) database (https://www.qiagenbioinformatics.com/products/ingenuity-pathway-analysis; accessed on 1 November 2021).

#### 2.5.4. Identification of Protein Predictors of EoT

To identify a set of proteins that best predict EoT, we performed elastic-net regularized logistic regression using the *glmnet* package in *R* (version 3.6.2). In the regression model, there are two user-defined hyper-parameters: elastic-net penalty, α, and tuning parameter, λ [34]. We chose an α that produced the highest prediction accuracy and a λ that provided the most regularized model such that the error is within one standard error of the minimum (usually denoted by *lambda.1se*). The optimal value of λ was obtained from leave-one-out cross-validation (LOOCV). To account for the relatively small sample size a bootstrap procedure was executed to obtain optimism-corrected performance metrics [35]. For details, see Section 5.3.4 of Clinical Prediction Models by Steyerberg and Ewout, 2019 [36]. Performance metrics included area under the curve (AUC), prediction accuracy, Brier scores, sensitivity, and specificity. No covariate for adjustment was considered. Proteins with non-zero coefficients were reported as strong predictors.

#### 2.5.5. Identification of Intracellular Proteins Associated with End-Organ Damage

Intracellular proteins differentially expressed between EoT and non-EoT groups and expressed within organs of interest were evaluated. Correlation coefficients were generated between MS-derived plasma protein abundance for proteins preferentially expressed in the liver and serum liver aminotransferases. An analysis of variance test was used to evaluate associations between MS-derived proteins primarily expressed in the kidney and acute kidney injury development. Acute kidney injury was defined by stage 2 or 3 Kidney Disease Improving Global Outcomes (KDIGO) criteria using a Modification of Diet in Renal Disease-derived reference for creatinine described previously by Hatton et al. [37].

## 3. Results

### 3.1. Clinical Characteristics

One subject was excluded for inadequate plasma sample, leaving 99 subjects for study inclusion. The EoT group consisted of 62 subjects with plasma Syn-1 ≥ 40 ng/mL, while the 37 remaining subjects were considered non-EoT. Patient demographic information, clinical data, and laboratory values are summarized in Table 1. EoT subjects were more severely injured, had more severe chest and abdominal injuries, were less likely to have a mechanism of injury related to burns, and were more likely to have blunt and penetrating trauma compared to non-EoT subjects. The EoT group was associated with a lower Glasgow coma scale score and systolic blood pressure on emergency department arrival. EoT patients had increased serum creatinine and glucose concentrations, increased base deficit, and decreased pH. Thromboelastography (TEG) demonstrated associations between EoT and increased activated clotting time, increased K-time, decreased α-angle, decreased maximum amplitude, and decreased G-value. EoT subjects required increased blood product transfusion. The EoT group had increased mortality within 72 h of admission; however, there was no statistically significant difference in overall in-hospital mortality between those with and without EoT. EoT patients were more likely to develop pneumonia and pulmonary embolism.

### 3.2. ELISA

ELISA data are summarized in Table 2. Plasma epinephrine, norepinephrine, and sTM concentrations were increased in the EoT group.

### 3.3. MS Proteomics

**Figure 1:**
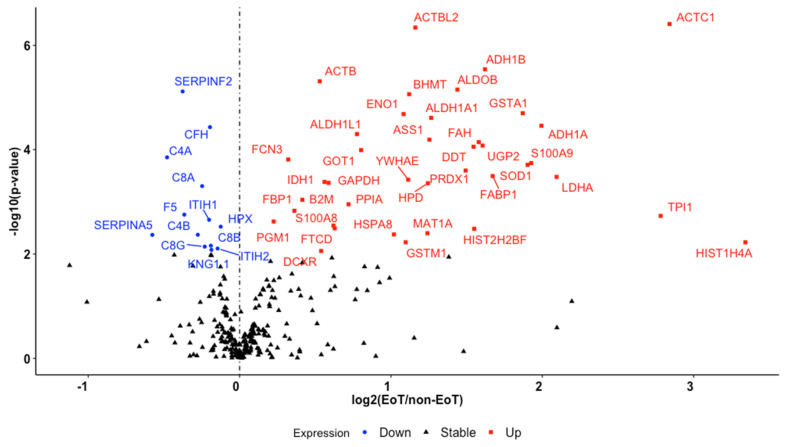
Volcano plot featuring differentially expressed proteins between the EoT and non-EoT group. EoT: endotheliopathy of trauma defined by plasma syndecan-1 ≥ 40 ng/mL; *p*-value: Benjamini and Hochberg false discovery rate-adjusted *p*-value; labeled proteins represent an adjusted *p*-value < 0.05.

Six hundred and five proteins were identified by MS for 99 trauma subjects with sufficient plasma samples. Four hundred and seventy-six proteins qualified as high confidence reads with an FDR < 0.01. The remaining 129 proteins were excluded. One hundred and seventy-eight proteins with missing values for >30% of subjects were omitted leaving 298 acceptable proteins. Eight proteins with >10 extreme outliers were then excluded resulting in 290 proteins for subsequent analysis. Fifty-two differentially expressed proteins between EoT and non-EoT groups were identified using a Benjamini and Hochberg FDR-adjusted *p*-value at 5%, including 39 upregulated proteins and 13 downregulated proteins associated with EoT. This information is summarized in Figure 1 and Table 3. The top ten proteins out of 42 identified by elastic-net regularized logistic regression with bootstrapping that best predict EoT in descending order included ACTC1, ADH1B, GSTA1, C4A, S100A9, HIST1H4A, ACTBL2, SERPINF2, CAT, and CFH. The regression model produced a prediction accuracy of 0.71 (95% CI: 0.62–0.78), a sensitivity of 0.81, a specificity of 0.70, and an area under the curve of 0.84 (95% CI: 0.80–0.89). Within the 52 proteins differentially expressed in the EoT group, ADH1A, MAT1A, BHMT, HPD, and FABP1 were selected for further analysis based on intracellular hepatocyte expression [38,39]. Serum liver transaminase data within three days of admission were available for 51 of the 99 patients. Each of the five hepatocyte proteins displayed statistically significant positive correlation coefficients between plasma protein abundance and serum aspartate transaminase (AST) and alanine aminotransferase (ALT) concentration, see Table 4. BHMT, HPD, and FABP1 were selected based on known intracellular kidney abundance [38,39]. None of these proteins demonstrated a statistically significant association with acute kidney injury defined by KDIGO criteria, see Table 5.

## 4. Discussion

Associations between EoT and clinical characteristics are consistent with previous studies for many parameters, including increased injury severity [17,18], hypotension [14,18], increased base deficit [17], hypocoaguable TEG profile [17], increased blood transfusion requirement [17,18], and increased early mortality [17,18]. A statistically significant increase in early death was observed in the EoT group, but not in overall in-hospital mortality. The majority of the burn patients were within the non-EoT group, many of whom succumbed to their injuries later in their hospital course which may contribute to this finding. The ELISA data is in agreement with previously reported associations between endothelial activation and sympathoadrenal hyperactivation, as increased serum concentrations of sTM [14,17,18], epinephrine [17], and norepinephrine [17] were observed in the EoT group. The 39 upregulated and 13 downregulated proteins that differentiate EoT from non-EoT have several significant clinical correlations.

### 4.1. Damage Associated Molecular Patterns

Four prominent damage-associated molecular pattern (DAMP) proteins were found to have elevated concentrations in the EoT group, including S100A8, S100A9, HIST1H4A, and PPIA. Protein S100A8 and protein S100A9, also known as myeloid-related protein-8 (MRP8) and myeloid-related protein-14 (MRP14), respectively, as well as the S100A8/A9 heterodimer known as calprotectin, represent mediators of acute inflammation [40]. When secreted these proteins induce leukocyte activation and chemotaxis through toll-like receptor 4 (TLR4) and receptor for advanced glycation end products (RAGE) pattern recognition receptors [40]. These pleiotropic proteins possess anti-inflammatory properties as well which may be suppressed by oxidative stress [41,42,43,44,45,46,47,48].

Histone H4 (HIST1H4A) is a regulator of chromatin packing and gene expression within cell nuclei [49]. However, this protein functions as a potent alarmin when introduced into circulation following cellular damage [50]. Histones induce cytokine release through interactions with toll-like receptors [51,52,53]. Extracellular histones represent the predominant contributor of cytotoxicity within neutrophil extracellular traps (NETs) [54], a network of molecules with thrombotic and antimicrobial functions involved in the innate immune response [55]. Glycocalyx constituents, including glycosaminoglycans and heparin, inhibit the cytotoxic effects of extracellular histones [56,57]. Circulating inter-α-trypsin inhibitor (ITIH1) is involved in histone sequestration in states of acute inflammation [58]. We find in the current EoT cohort a diminished abundance of circulating ITIH1 paired with increased plasma histone H4. Elevated circulating histone H4 specifically has been observed in trauma subjects previously [59,60], and among all histone components, H4 is considered the most cytotoxic and the greatest contributor to fibrinolysis [61,62].

Cyclophilin A, also known as peptidyl-prolyl cis-trans isomerase A, CYPA, or PPIA, is a ubiquitous intracellular protein that is released into circulation by vascular smooth muscle cells in response to oxidative stress [63]. This protein can also be released by endothelial cells [64], platelets [65], and macrophages [66]. PPIA promotes cytokine release through interactions with cell surface receptor CD147 [67].

A summary of previously established associations between these DAMPs and pathophysiology relevant to the systemic response to trauma is summarized in Table 6. DAMPs represent a potential driver of ongoing endothelial injury and activation, as well as attenuation of the permeability barrier. In addition to the physiologic challenges associated with hyper-permeable vessels, these proteins contribute to organ injury through direct cytotoxic effects and microvascular thrombosis. This EoT phenotype likely feeds self-amplifying loops involving sympathoadrenal hyperactivation and cytokine release [68]. DAMPs have been implicated in the etiology of immune failure and immune-mediated host injury in trauma subjects [69,70]. A Danger Model has been proposed based on the assertion that tissue damage governs activation of the immune system, rather than an innate sense of self versus non-self [71,72,73]. DAMPs likely represent the source of immune-mediated host injury and attenuated host immune defense in a manner analogous to the nonspecific activation and subsequent exhaustion of coagulation factors in the acute coagulopathy of trauma. DAMP-induced damage can, in turn, generate DAMPs and precipitate self-perpetuating physiologic disarray in injured patients. A variety of potential pharmacological therapeutics exist with the potential to arrest these DAMP-related pathophysiological processes in trauma [54,74,75,76,77,78,79,80,81,82,83,84,85,86].

### 4.2. End-Organ Damage

Several proteins demonstrating an increased abundance in the EoT group are suggestive of end-organ damage. Actin α cardiac muscle-1 (ACTC1) represents the single best predictor of EoT of the 290 MS-derived proteins determined by elastic net regularized regression. ACTC1 is typically an intracellular protein and represents the major form of actin within cardiac sarcomeres [38,161,162,163], although it may also be found in smooth muscle and skeletal muscle [39,164,165,166]. Elevated serum ACTC1 has been associated with endotoxemia, potentially due to sarcomere disruption [167]. There is little data available regarding the molecular and clinical significance of circulating ACTC1. However, its characteristics can be inferred based on similarities to comparable proteins including troponin and other subtypes of actin. Circulating α-actin has been utilized as an early biomarker reflecting cardiac damage secondary to myocardial infarction in a manner similar to troponin [168]. Elevated circulating troponin has been used to diagnose secondary cardiac injury without related thoracic injury in trauma cohorts, even in younger patients unlikely to have had pre-injury cardiac disease [169]. This trauma-induced secondary cardiac injury has been associated with mortality [169,170,171]. The etiology underlying cardiac impairment is believed to involve DAMP and pro-inflammatory mediators as well as catecholamine surge resulting in cardiomyocyte cytotoxicity and dysfunction [169,172,173,174]. Histologic changes in cardiomyocytes identified in trauma patients upon autopsy include congestion, interstitial edema, and myofibril degeneration [175,176]. Similar sarcomere disorganization has been observed in murine models of hemorrhagic shock [177].

In the present study an association between increased plasma concentration of DAMPs, epinephrine, norepinephrine, and EoT is observed. Taken together, the elevated abundance of ACTC1 may reflect DAMP and catecholamine-induced cardiomyocyte damage with subsequent leakage of myofilament components into plasma. The hypotension and metabolic acidosis associated with EoT may signify increased cardiac demand and heart strain contributing to the release of ACTC1 into circulation. In addition to representing a biomarker for cardiac damage, ACTC1 itself may contribute to poor outcomes in EoT. Circulating actin is cytotoxic to endothelial cells [178], impairs clearance of cytotoxic cell-free circulating DNA [179,180], promotes coagulopathy [181], and disrupts host defenses [182]. Exhaustion of the actin-scavenger system is a risk factor for mortality in trauma [183,184].

A statistically significant increase in pulmonary embolism (PE) and pneumonia was observed in the EoT group. Contemporary guidelines recommend pharmaceutical venous thromboembolism (VTE) prophylaxis in the majority of moderate to high-risk trauma patients [185]. Even with perfect adherence to prophylaxis guidelines breakthrough VTE following injury persists [186,187], particularly in those with severe injuries [188]. Breakthrough cases of PE in trauma appear to have unique characteristics and an etiology distinct from other forms of VTE. These blood clots are more likely to arise in the segmental and sub-segmental vasculature [189] and appear to arise de novo within the lung rather than from proximal pelvic vein embolization [189,190,191]. Several EoT-associated DAMPs are cytotoxic to the pulmonary endothelium and capable of inciting microthrombosis within the pulmonary vasculature. These proteins may be contributing to the etiology of breakthrough PE, as well as pneumonia, within the EoT group. There was no statistically significant difference in respiratory failure or ARDS development between the EoT and non-EoT groups. This may be related to insufficient sample size and censoring secondary to increased early mortality in the EoT group. Of note, both elevated ACTC1 and increased incidence of PE could be related to direct injury to the heart and lungs, respectively, as the EoT group demonstrated increased chest abbreviated injury score compared to non-EoT subjects.

An increased abundance of plasma fatty-acid binding protein 1 (liver type; FABP1) was observed in the EoT group. This protein is expressed in the intestines, liver, and kidney [38,39,192], and has many intracellular functions including antioxidant defense [193]. Elevated circulating FABP1 is associated with severe abdominal injury and acute renal failure in trauma subjects [194,195]. Increased plasma FABP1 may result from direct organ damage or ischemia [196,197,198,199,200]. The EoT group was associated with increased abdominal abbreviated injury score and increased blood transfusion requirement, suggesting both direct abdominal organ injury and hypoperfusion. Plasma FABP1 is positively correlated with serum transaminases indicating FABP1 leakage from hepatocytes. However, this does not preclude additional concurrent plasma FABP1 contributions from the kidney and the intestines. FABP1 possesses heme-binding and antioxidant properties [193], characteristics that may provide some benefit to the acutely injured trauma patient. This may represent an evolved adaptive response to oxidative damage in severe injury as the superoxide dismutase SOD1, a primarily intracellular antioxidant [201], is also elevated in the plasma of EoT subjects.

The EoT group was associated with increased plasma concentrations of four additional plasma proteins that are primarily expressed in hepatocytes and renal cells. ADH1A (alcohol dehydrogenase 1A) and MAT1A (methionine adenosyltransferase 1A) are preferentially expressed in the liver [38,39]. BHMT (betaine-homocysteine S-methyltransferase) and HPD (4-hydroxyphenylpyruvate dioxygenase) are principally expressed in both the liver and kidneys [38,39]. ADH1A, MAT1A, BHMT, and HPD each demonstrated a statistically significant positive correlation coefficient with serum AST and ALT concentration. There was no statistically significant association observed between BHMT or HPD and acute kidney injury. These findings suggest an association between liver injury, leakage of hepatocyte proteins into circulation, and EoT.

Elevated plasma BHMT may indicate underlying metabolic processes related to EoT. Increased abundance of intracellular liver proteins in circulating plasma is likely a function of both hepatocyte leakage and hepatocyte intracellular protein concentration. BHMT is a regulator of homocysteine metabolism and methionine biosynthesis through the facilitation of betaine methyl group donation [202]. BHMT expression is upregulated by the amino acid taurine [203], an osmolyte with important physiologic functions involving resistance to osmotic stress and maintenance of microvascular flow [204,205,206,207]. Increased plasma BHMT in the EoT group may reflect increased taurine abundance within hepatocytes with subsequent BHMT upregulation in addition to liver damage. This finding may denote a compensatory metabolic response in EoT subjects.

### 4.3. Coagulation

Coagulation factor V (FV) was the only coagulation factor with a statistically significant decrease in plasma abundance in EoT compared to non-EoT. EoT was associated with a hypocoaguable TEG profile, consistent with previous findings demonstrating an association between lower levels of circulating FV and acute coagulopathy following trauma [208,209,210,211,212,213,214]. A proportion of plasma FV undergoes endocytosis and is sequestered within platelet alpha granules [215,216,217,218], and this intra-platelet FV is integral for achieving hemostasis following traumatic hemorrhage [219,220]. The factor V Leiden (FVL) mutation results in an FV phenotype that is resistant to proteolysis by activated protein C (APC) [221] and represents the most common inherited form of thrombophilia in Caucasians [222]. The high frequency of this polymorphism is believed to be due to a conferred evolutionary advantage secondary to improved hemostasis in states of hemorrhage, including trauma [223,224,225]. This theory is supported by observations of decreased peri-partum bleeding in FVL carriers [226,227,228]. The effect of FVL on bleeding and nonbleeding traumatic injury is unknown. The influence of FVL on the function of APC, which is involved in many processes relevant to trauma including the degradation of extracellular histones [52,53,54,92], is not fully elucidated. However, FV may be the only coagulation factor deficit that can be identified at the scene of injury [209], is the most consistent coagulation factor deficiency observed on trauma admission blood samples [211], and regulators of FV abundance appear to be distinct from those involved in other coagulation proteins [213]. It may not be a coincidence that the strongest genotype candidate for improved outcomes in traumatic bleeding involves conservation of FV, the same coagulation factor deficiency that is most directly tied to the acute coagulopathy of trauma and endotheliopathy. Associations between EoT and increased blood transfusion requirements, decreased plasma FV, coagulopathy, and elevated plasma DAMPs reinforce the suspicion that FV may serve an important role in the systemic response to trauma.

We report decreased plasma concentration of α-2-plasmin inhibitor (SERPINF2; AAP) and plasminogen activator inhibitor (SERPINA5; PAI-1) in the EoT group. These proteins represent inhibitors of fibrinolysis and depletion of circulating AAP and PAI-1 is associated with the acute coagulopathy of trauma [229], and hyperfibrinolysis [230,231,232,233]. The primary driver of trauma-related hyperfibrinolysis is postulated to be insufficient PAI-1 concentration relative to increased tissue plasminogen activator (tPA) release by the microvascular endothelium [234]. Decreased AAP and PAI-1 indicates fibrinolysis within the endotheliopathy group despite the lack of an observed difference in TEG clot lysis between EoT and non-EoT in the present study. Varying degrees of fibrinolysis exist within trauma subjects [233], and a spectrum of coagulation phenotypes may be present within EoT as well. Despite this potential heterogeneity within the EoT group, decreased inhibitors of fibrinolysis and a hypocoaguable coagulation profile are suggestive of consumptive coagulopathy. These findings may represent widespread microthrombosis provoked by endothelial injury and DAMPs. Of note, a previous study has failed to identify a significant association between PAI-1 level and syndecan-1 shedding [27]. However, these findings were based on a higher plasma syndecan-1 concentration cutoff and a smaller sample size.

Decreased plasma high-molecular-weight kininogen (HMWK; KNG1) was associated with the EoT group. HMWK maintains an antithrombotic endothelial cell surface and patent blood flow within the microvasculature [235,236,237,238]. HMWK cleavage by factor XII (Hageman Factor) initiates the coagulation cascade and conversion of prekallikrein to kallikrein, subsequent release of bradykinin, and increased permeability and vasodilation [238,239,240]. This process can be incited by complement, neutrophils, and tPA [235,241,242]. Glycosaminoglycans on the endothelial surface preserve HMWK, and degradation of the glycocalyx results in HMWK proteolysis and bradykinin-mediated attenuation of the permeability barrier [243]. Consumption of HMWK indicates thrombosis formation and increased permeability secondary to glycocalyx deterioration.

Hemopexin (HPX) is a circulating free heme carrier protein that neutralizes oxidative damage and transports heme to the liver [244,245]. Hemopexin reserve can be diminished following traumatic hemorrhage and blood product resuscitation, and depletion is associated with increased vascular permeability and susceptibility to infection [246]. Decreased hemopexin abundance implies red blood cell lysis within the EoT group and is likely related to consumptive coagulopathy and red blood cell transfusion.

### 4.4. Complement

In the EoT group, we find decreased abundance of complement inhibitor CFH (complement factor H), complement activator FCN3 (ficolin-3), and complement components C8A, C8B, C8G, C4A, and C4B. CFH binds heparin and glycosaminoglycan linkages on endothelial cell surfaces and supports endothelial integrity by suppression of nonspecific alternative complement activation [247,248]. Mutations in the endothelial binding domain of CFH can manifest as an atypical hemolytic uremic syndrome (aHUS) resulting in alternative complement pathway-induced endothelial damage [248]. Endotheliopathy is believed to serve as the initiator of aHUS by provoking arteriole and capillary wall thickening, intraluminal platelet activation, microthrombosis, vessel occlusion, and end-organ damage [249]. Factor H provides specificity to alternative complement activation [250], and a deficiency of this protein within the EoT group may allow for unregulated complement-induced cytotoxicity of host endothelium and a clinical phenotype similar to aHUS.

Ficolin-3 is a known activator of the lectin complement pathway [251]. Cleavage of C4 into C4a and C4b is involved in the initiation of the classic and lectin complement pathways [252], while C8 is a constituent of the terminal complement complex. C4a is a potent anaphylatoxin capable of inducing endothelial activation [253], as is the terminal complement complex [254,255,256,257,258,259,260]. Complement activation following trauma has been associated with increased injury severity and red blood cell and platelet dysfunction [261,262,263].

Reduced plasma abundance of these complement components within EoT subjects, particularly the deficiency of CFH, is suggestive of nonspecific complement activation, widespread deposition of complement components into host endothelium, and subsequent exhaustion of the complement system. This process likely contributes to increased endothelial permeability, coagulopathy, and end-organ damage.

### 4.5. Limitations

The limitations of this study include those inherent to MS methodology, as no single proteomic assay can identify all proteins within a plasma sample. For this reason, proteins significant to EoT may not be included in this analysis, particularly low-abundance plasma proteins which can be better evaluated through a high abundance protein depletion step prior to MS. This data set is restricted by sample size which was not sufficient to accurately identify subgroups within EoT subjects. Evaluation of sub-phenotypes within EoT may allow for a more personalized approach in trauma management, as this strategy has shown promise in similar heterogeneous pathologies including ARDS [264,265]. Further, some relevant proteins may not yield significant associations with EoT due to type II error. Finally, we did not normalize total protein concentration to control for hemodilution. This method has been utilized in similar proteomic studies and may provide distinct information [159].

## 5. Conclusions

The clinical significance of endothelial permeability has been debated since early dog models of hemorrhagic shock in the 1940s [266,267]. Shedding of the glycocalyx does not imply endothelial cell death, but rather reflects endothelial activation and increased permeability [13,31]. This is an important distinction, as these dysfunctional living cells represent an opportunity for intervention that would not exist in nonviable endothelial cells. Within the proteomic profile of EoT, we present several DAMPs that contribute to endothelial activation, coagulopathy, and host tissue destruction. We report signs of non-specific coagulation and complement activation, as well as the exhaustion of vital regulatory components within these systems. We describe circulating intracellular proteins suggestive of end-organ damage. The proteomic profile of EoT reflects mechanisms evolutionarily intended for local tissue injury amplified to a pathologic level. This secondary injury represents a perpetual source of damage in itself and current management practices in trauma largely allow this process to continue unabated. The key to improving long-term outcomes in trauma depends on the mitigation of this secondary traumatic insult which may require a multimodal approach. Herein, we have identified proteins and pathways that are likely to be driving the pathologic processes underlying EoT which serve as targets for pharmacological therapeutics.

## Figures and Tables

**Table 1:** Demographics, clinical data, laboratory values and outcomes of trauma patients by EoT status. Data are presented as the median (IQR) for continuous variables and *n* (%) for categorical and ordinal variables.

	*n*	All Trauma Subjects (*n* = 99)	*n*	Non-EoT (*n* = 37)	*n*	EoT (*n* = 62)	*p*-Value
***Demographics***							
Age (years)	99	44 (34, 54)	37	46 (36, 58)	62	44 (32, 51)	0.265
Body mass index	96	27.95 (25, 31)	37	28.3 (25, 31)	59	27.2 (25, 31)	0.698
Male	99	73 (74%)	37	25 (68%)	62	48 (77%)	0.347
Race	99		37		62		0.616
White		70 (71%)		42 (67.74%)		28 (75.68%)	
African American		27 (27%)		9 (24%)		18 (29%)	
Asian/Pacific Islander		2 (2%)		1 (2.7%)		1 (1.6%)	
Ethnicity	99		37		62		1
Hispanic		23 (24%)		8 (22%)		15 (25%)	
***Injury Characteristics***							
Injury severity score	99	25 (16, 29)	37	22 (9, 25)	62	26 (22, 34)	<0.001 *
Head AIS		0 (0, 0)		0 (0,0)		0 (0,0)	0.907
Face AIS		0 (0, 0)		0 (0,0)		0 (0,0)	0.154
Chest AIS		3 (0, 4)		0 (0, 3)		3 (0,4)	0.026 *
Abdomen AIS		0 (0, 4)		0 (0, 2)		2 (0, 4)	0.001 *
Extremity AIS		0 (0, 3)		0 (0, 3)		0 (0, 3)	0.177
External AIS		1 (0, 2)		1 (0, 3)		1 (1, 2)	0.188
Mechanism of injury	99		37		62		0.005 *
Blunt		55 (56%)		16 (43%)		39 (63%)	
Penetrating		25 (25%)		8 (22%)		17 (27%)	
Burn		19 (19%)		13 (35%)		6 (10%)	
***Admission Vital Signs and Labs***							
Glasgow Coma Scale	99		37		62		0.018 *
3		37 (37.4%)		10 (27.0%)		27 (43.5%)	
7		2 (2.0%)		0 (0.0%)		2 (3.2%)	
8		1 (1.0%)		0 (0.0%)		1 (1.6%)	
9		2 (2.0%)		0 (0.0%)		2 (3.2%)	
11		1 (1.0%)		0 (0.0%)		1 (1.6%)	
13		2 (2.0%)		1 (2.7%)		1 (1.6%)	
14		5 (5.1%)		1 (2.7%)		4 (6.5%)	
15		49 (49.5%)		25 (67.6%)		24 (38.7%)	
Systolic BP (mm Hg)	97	118 (99, 137)	35	125 (113, 139)	62	110 (97, 130)	0.018
Heart rate (bpm)	97	100 (83, 116)	35	96 (79, 110)	62	101 (84, 121)	0.252
Creatinine (mg/dL)	97	1.21 (1.00, 1.47)	37	1.08 (0.95, 1.30)	60	1.32 (1.01, 1.57)	0.003 *
Glucose (mg/dL)	97	163 (121, 213)	37	153 (117, 180)	60	180 (133, 230)	0.020 *
Albumin (g/dL)	34	3.10 (2.73, 3.40)	13	3.00 (2.90, 3.50)	21	3.10 (2.70, 3.40)	0.845
Base excess (mmol/L)	98	−6 (−9, −2)	37	−2 (−6, −2)	61	−8 (−12, −4)	<0.001 *
pH	96	7.26 (7.18, 7.35)	36	7.34 (7.28, 7.35)	60	7.20 (7.10, 7.28)	<0.001 *
Platelet count (K/uL)	95	230 (187, 281)	37	242 (199, 314)	58	215 (164, 277)	0.127
ACT (s)	91	113 (105, 121)	34	105 (105, 113)	57	113 (105, 121)	0.047 *
TEG R-time (min)	91	0.7 (0.6, 0.8)	34	0.6 (0.6, 0.7)	57	0.70 (0.6, 0.8)	0.091
TEG K-time (min)	90	1.30 (1.10, 1.80)	34	1.20 (1.02, 1.50)	56	1.45 (1.20, 1.80)	0.023 *
TEG α-angle (degrees)	91	74 (70, 77)	34	76 (72,78)	57	73 (69, 76)	0.016 *
TEG maximum amplitude (mm)	91	64 (59, 68)	34	66 (62, 69)	57	62 (58, 66)	0.002 *
TEG G-value (Kdynes/cm^2^)	91	8.70 (7.15, 10.50)	34	9.75 (8.03, 11.30)	57	8.20 (6.80, 9.50)	0.002 *
TEG estimated lysis (%)	91	1 (0.1, 2.4)	34	1.2 (0.2, 2.5)	57	0.9 (0.1, 2.5)	0.961
***Transfusions***							
Any blood product prehospital to 24 h	99	71 (72%)	37	21 (57%)	62	50 (81%)	0.020 *
***Outcomes***							
ICU-free days (30 days)	99	19 (0, 28)	37	26 (0, 30)	62	9 (0, 27)	0.058
ICU days	99	3, (0, 11)	37	2 (0, 10)	62	4 (0, 13)	0.450
12-h mortality	99	13 (13%)	37	1 (3%)	62	12 (19%)	0.028 *
24-h mortality	99	14 (14%)	37	1 (3%)	62	13 (21%)	0.015 *
72-h mortality	99	15 (15%)	37	1 (3%)	62	14 (23%)	0.008 *
In-hospital mortality	99	37 (37%)	37	10 (27%)	62	27 (44%)	0.133
***Complications***							
Acute renal failure	99	27 (27%)	37	10 (27%)	62	17 (27%)	1
Deep vein thrombosis	99	1 (1%)	37	1 (3%)	62	0 (0%)	0.374
Pulmonary embolism	99	7 (7%)	37	0 (0%)	62	7 (11%)	0.043 *
Pneumonia	99	16 (16%)	37	2 (5%)	62	14 (23%)	0.026 *
Sepsis	99	19 (19%)	37	7 (19%)	62	12 (20%)	1
Urinary tract infection	99	6 (6%)	37	4 (11%)	62	2 (3%)	0.193
ARDS	99	6 (6%)	37	3 (8%)	62	3 (5%)	0.668
Respiratory failure	99	40 (40%)	37	14 (38%)	62	26 (42%)	0.833
SIRS	99	7 (7%)	37	4 (11%)	62	3 (5%)	0.419
Decubitus ulcer	99	3 (3%)	37	0 (0%)	62	3 (5%)	0.291
Multiple organ failure	99	17 (17%)	37	7 (19%)	62	10 (16%)	0.786

EoT: endotheliopathy of trauma defined by plasma syndecan-1 ≥ 40 ng/mL; AIS: abbreviated injury scale; TEG: thromboelastography; ICU: intensive care unit; ARDS: acute respiratory distress syndrome; SIRS: systemic inflammatory response syndrome; * *p*-value < 0.05.

**Table 2:** Plasma enzyme linked immunosorbent assay results presented as median (IQR).

	*n*	All Trauma Subjects (*n* = 99)	Non-EoT (*n* = 62)	EoT (*n* = 37)	*p*-Value
Epinephrine	99	315 (140, 906)	241 (89, 392)	458 (201, 2136)	0.004 *
Norepinephrine	99	1295 (595, 2432)	780 (388, 1308)	1799 (803, 3642)	<0.001 *
Soluble Thrombomodulin	99	6.6 (5.2, 9.5)	5.2 (4.7, 6.5)	7.5 (6.1, 11.4)	<0.001 *
Syndecan-1	99	67 (28, 184)	25 (19, 31)	165 (79, 195)	<0.001 *

EoT: endotheliopathy of trauma defined by plasma syndecan-1 ≥ 40 ng/mL; biomarker concentrations are reported in ng/mL; * *p*-value < 0.05.

**Table 3:** Differentially expressed plasma proteins between EoT and non-EoT groups based on adjusted *p*-value < 0.05.

Gene Symbol	Protein Description	Raw *p*-Value ^¥^	Adjusted *p*-Value ^£^	Fold Change ^±^
HIST1H4A	Histone H4	5.97 × 10^−3^	3.69 × 10^−2^	3.34
ACTC1	Actin, alpha cardiac muscle 1	3.90 × 10^−7^	6.61 × 10^−5^	2.84
TPI1	Isoform 2 of triosephosphate isomerase	1.86 × 10^−3^	1.54 × 10^−2^	2.78
LDHA	L-lactate dehydrogenase A chain	3.33 × 10^−4^	3.86 × 10^−3^	2.09
ADH1A	Alcohol dehydrogenase 1A	3.48 × 10^−5^	8.94 × 10^−4^	1.99
S100A9	Protein S100-A9	1.80 × 10^−4^	2.49 × 10^−3^	1.93
SOD1	Superoxide dismutase	1.95 × 10^−4^	2.58 × 10^−3^	1.90
GSTA1	Glutathione S-transferase	2.00 × 10^−5^	6.71 × 10^−4^	1.87
FABP1	Fatty acid-binding protein, liver	3.20 × 10^−4^	3.86 × 10^−3^	1.67
ADH1B	Alcohol dehydrogenase 1B	2.87 × 10^−6^	2.78 × 10^−4^	1.62
UGP2	UTP--glucose-1-phosphate uridylyltransferase	8.35 × 10^−5^	1.50 × 10^−3^	1.61
FAH	Fumarylacetoacetase	7.17 × 10^−5^	1.39 × 10^−3^	1.58
HIST2H2BF	Histone H2B type 2-F	3.29 × 10^−3^	2.33 × 10^−2^	1.55
DDT	D-dopachrome decarboxylase	8.78 × 10^−5^	1.50 × 10^−3^	1.55
PRDX1	Peroxiredoxin-1	2.51 × 10^−4^	3.17 × 10^−3^	1.49
ALDOB	Fructose-bisphosphate aldolase B	7.04 × 10^−6^	3.58 × 10^−4^	1.44
ALDH1A1	Retinal dehydrogenase 1	2.46 × 10^−5^	7.12 × 10^−4^	1.27
ASS1	Argininosuccinate synthase	6.42 × 10^−5^	1.33 × 10^−3^	1.25
HPD	4-hydroxyphenylpyruvate dioxygenase	4.40 × 10^−4^	4.40 × 10^−3^	1.24
MAT1A	S-adenosylmethionine synthase isoform type-1	3.99 × 10^−3^	2.75 × 10^−2^	1.24
ACTBL2	Beta-actin-like protein 2 O	4.56 × 10^−7^	6.61 × 10^−5^	1.16
BHMT	Betaine--homocysteine S-methyltransferase 1	8.64 × 10^−6^	3.58 × 10^−4^	1.12
YWHAE	14-3-3 protein epsilon	3.77 × 10^−4^	4.20 × 10^−3^	1.11
GSTM1	Glutathione S-transferase Mu 1	5.97 × 10^−3^	3.69 × 10^−2^	1.10
ENO1	Alpha-enolase	2.08 × 10^−5^	6.71 × 10^−4^	1.08
HSPA8	Heat shock cognate 71 kDa protein	4.22 × 10^−3^	2.77 × 10^−2^	1.02
GOT1	Aspartate aminotransferase, cytoplasmic	1.02 × 10^−4^	1.64 × 10^−3^	0.80
ALDH1L1	Cytosolic 10-formyltetrahydrofolate dehydrogenase	5.02 × 10^−5^	1.12 × 10^−3^	0.78
PPIA	Peptidyl-prolyl cis-trans isomerase A	1.11 × 10^−3^	1.01 × 10^−2^	0.72
FTCD	Formimidoyltransferase-cyclodeaminase	3.22 × 10^−3^	2.33 × 10^−2^	0.63
S100A8	Protein S100-A8	2.85 × 10^−3^	2.17 × 10^−2^	0.62
GAPDH	Glyceraldehyde-3-phosphate dehydrogenase	4.33 × 10^−4^	4.40 × 10^−3^	0.59
IDH1	Isocitrate dehydrogenase [NADP] cytoplasmic	4.15 × 10^−4^	4.40 × 10^−3^	0.56
DCXR	L-xylulose reductase	8.75 × 10^−3^	4.88 × 10^−2^	0.54
ACTB	Actin, cytoplasmic 1	4.90 × 10^−6^	3.55 × 10^−4^	0.53
B2M	Beta-2-microglobulin	9.11 × 10^−4^	8.52 × 10^−3^	0.41
FBP1	Fructose-1,6-bisphosphatase 1	1.48 × 10^−3^	1.30 × 10^−2^	0.36
FCN3	Ficolin-3 OS = Homo sapiens	1.54 × 10^−4^	2.24 × 10^−3^	0.32
PGM1	Phosphoglucomutase-1	2.38 × 10^−3^	1.87 × 10^−2^	0.23
HPX	Hemopexin	2.99 × 10^−3^	2.22 × 10^−2^	−0.12
ITIH2	Inter-alpha-trypsin inhibitor heavy chain H2	7.85 × 10^−3^	4.55 × 10^−2^	−0.15
KNG1	Kininogen-1	8.33 × 10^−3^	4.73 × 10^−2^	−0.19
C8B	Complement component C8 beta chain	6.92 × 10^−3^	4.18 × 10^−2^	−0.19
CFH	Complement factor H	3.70 × 10^−5^	8.94 × 10^−4^	−0.20
ITIH1	Inter-alpha-trypsin inhibitor heavy chain H1	2.21 × 10^−3^	1.78 × 10^−2^	−0.20
C8G	Complement component C8 gamma chain	7.23 × 10^−3^	4.28 × 10^−2^	−0.23
C8A	Complement component C8 alpha chain	4.99 × 10^−4^	4.82 × 10^−3^	−0.25
C4B	Complement C4-B	4.27 × 10^−3^	2.77 × 10^−2^	−0.28
F5	Coagulation factor V	1.76 × 10^−3^	1.50 × 10^−2^	−0.37
SERPINF2	Alpha-2-antiplasmin	7.66 × 10^−6^	3.58 × 10^−4^	−0.38
C4A; C4B	Complement C4-A	1.40 × 10^−4^	2.14 × 10^−3^	−0.48
SERPINA5	Plasma serine protease inhibitor	4.30 × 10^−3^	2.77 × 10^−2^	−0.58

EoT: endotheliopathy of trauma defined by plasma syndecan-1 ≥ 40 ng/mL; ^¥^ raw *p*-value based on a two-sided *t*-test; ^£^ Benjamini and Hochberg false discovery rate-adjusted *p*-value; **^±^** fold-change based on EoT/non-EoT ratio.

**Table 4:** Correlation with 95% confidence interval between plasma abundance of upregulated intracellular hepatic proteins associated with the EoT group and serum transaminases.

	AST		ALT	
	r	*p*-Value	r	*p*-Value
BHMT	0.837 (0. 0.729, 0.904)	<0.001 *	0.894 (0.820, 0.938)	<0.001 *
ADH1A	0.907 (0. 842, 0.946)	<0.001 *	0.795 (0.666, 0.878)	<0.001 *
MAT1A	0.441 (0.188, 0.639)	0.001 *	0.310 (0.038, 0.539)	0.027 *
HPD	0.631 (0.430, 0.772)	<0.001 *	0.654 (0.461, 0.787)	<0.001 *
FABP1	0.828 (0.716, 0.899)	<0.001 *	0.732 (0.572, 0.838)	<0.001 *

EoT: endotheliopathy of trauma defined by plasma syndecan-1 ≥ 40 ng/mL; r: correlation coefficient; AST: aspartate transaminase; ALT: alanine aminotransferase; * *p*-value < 0.05; transaminase data were available for 51 subjects.

**Table 5:** Comparison between plasma abundance of upregulated intracellular renal proteins associated with the EoT group and acute kidney injury development presented as median (IQR).

	non-AKI *n* = 72	AKI *n* = 27	*p*-Value
BHMT	2,039,625 (1,498,128, 5,608,227)	1,276,029 (1,285,297, 2,118,322)	0.226
HPD	3,018,214 (2,053,260, 8,459,705)	3,118,767 (1,450,326, 5,226,505)	0.188
FABP1	3,362,220 (1,877,758, 16,830,020)	3,032,475 (2,430,624, 5,302,253)	0.997

EoT: endotheliopathy of trauma defined by plasma syndecan-1 ≥ 40 ng/mL; AKI: acute kidney injury defined by stage 2 or 3 Kidney Disease Improving Global Outcomes group criteria using a Modification of Diet in Renal Disease derived reference creatinine.

**Table 6:** Damage-associated molecular patterns with increased abundance in the EoT group and associations with pathophysiology relevant to the systemic response to trauma.

	S100-A8, S-100A9, S100-A8/A9	Histones	PPIA
Pro-inflammatory	[87,88,89,90,91]	[50,51,53,60,92]	[66,93,94]
Anti-inflammatory	[41,42,95,96,97]		
Thrombosis	[87,98,99,100,101]	[52,85,102,103,104,105,106,107]	[65,80,108]
Endothelial impairment and activation	[109,110,111]	[52,53,54,57,60,92,112]	[94,113,114,115]
Endothelial permeability	[87,116,117,118]	[56,118,119]	
Cardiac injury	[74,120,121,122,123]	[124,125,126]	[127,128,129,130]
Pulmonary injury	[52,131,132,133,134]	[56,60,135,136]	[64,137]
Renal injury	[138,139,140,141,142]	[52,143,144]	[145,146,147]
Hepatic injury	[76,148,149,150]	[50,51,151,152]	[153,154,155,156]
Trauma populations	- Post injury elevations in circulating S100-A8/9 [157] - Decreased rate of post-injury circulating S100-A8/9 elevation is associated with increased risk of infection [157]	- Increased injury severity [59] - Decreased admission Glasgow coma scale [158] - Increased sympatho-adrenal activation [59] - Increased inflammatory cytokine release [59] - Coagulopathy [25,59,60,106,159] - Increased ventilator days [158] - Multiple organ failure [158] - Acute lung injury [60,158] - Mortality [158]	- Mortality [160]

EoT: endotheliopathy of trauma defined by plasma syndecan-1 ≥ 40 ng/mL.

## Data Availability

The data that support the findings of this study are available from the corresponding author, J.D.K., upon reasonable request.
